# Non-invasive differentiation of idiopathic inflammatory myopathy with cardiac involvement from acute viral myocarditis using cardiovascular magnetic resonance imaging T1 and T2 mapping

**DOI:** 10.1186/s12968-018-0430-6

**Published:** 2018-02-12

**Authors:** Adrian T. Huber, Marine Bravetti, Jérôme Lamy, Tania Bacoyannis, Charles Roux, Alain de Cesare, Aude Rigolet, Olivier Benveniste, Yves Allenbach, Mathieu Kerneis, Philippe Cluzel, Nadjia Kachenoura, Alban Redheuil

**Affiliations:** 1 Sorbonne Universités, UPMC Univ Paris 06, INSERM 1146, CNRS 7371, Laboratoire d’Imagerie Biomédicale (LIB), Faculté de Médecine, 91, Boulevard de l’hôpital, 75013 Paris, France; 2 Department of Cardiovascular Imaging, Interventional and Thoracic Radiology, Institute of Cardiology, Hôpital Pitié-Salpêtrière, Paris, France; 3 Department of Diagnostic, Interventional and Pediatric Radiology, Inselspital, Bern University Hospital, University of Bern, Bern, Switzerland; 4 Institute of Cardiometabolism and Nutrition (ICAN), Paris, France; 5 Department of Internal Medicine, Hôpital Pitié-Salpêtrière, Paris, France; 6 Sorbonne Universités, UPMC Univ Paris 06, INSERM UMR974, Centre de Recherche en Myologie, Paris, France; 7 Department of Cardiology, Institute of Cardiology, Hôpital Pitié-Salpêtrière, Paris, France

**Keywords:** Cardiac inflammation, Systemic myositis, CMR T1/T2 mapping, Extracellular volume, Skeletal muscle

## Abstract

**Background:**

Idiopathic inflammatory myopathy (IIM) is a group of autoimmune diseases with systemic myositis which may involve the myocardium. Cardiac involvement in IIM, although often subclinical, may mimic clinical manifestations of acute viral myocarditis (AVM). Our aim was to investigate the usefulness of the combined analysis of cardiovascular magnetic resonance (CMR) T1 and T2 mapping parameters measured both in the myocardium and in the thoracic skeletal muscles to differentiate AVM from IIM cardiac involvement.

**Methods:**

Sixty subjects were included in this retrospective study (36 male, age 45 ± 16 years): twenty patients with AVM, twenty patients with IIM and cardiac involvement and twenty healthy controls. Study participants underwent CMR imaging with modified Look-Locker inversion-recovery (MOLLI) T1 mapping and 3-point balanced steady-state-free precession T2 mapping. Relaxation times were quantified after endocardial and epicardial delineation on basal and medial short-axis slices, as well as in different thoracic skeletal muscle groups present in the CMR field-of-view. ROC-Analysis was performed to assess the ability of mapping indices to discriminate the study groups.

**Results:**

Mapping parameters in the thoracic skeletal muscles were able to discriminate between AVM and IIM patients. Best skeletal muscle parameters to identify IIM from AVM patients were reduced post-contrast T1 and increased extracellular volume (ECV), resulting in an area under the ROC curve (AUC) of 0.95 for post-contrast T1 and 0.96 for ECV. Conversely, myocardial mapping parameters did not discriminate IIM from AVM patients but increased native T1 (AUC 0.89 for AVM; 0.84 for IIM) and increased T2 (AUC 0.82 for AVM; 0.88 for IIM) could differentiate both patient groups from healthy controls.

**Conclusion:**

CMR myocardial mapping detects cardiac inflammation in AVM and IIM compared to normal myocardium in healthy controls but does not differentiate IIM from AVM. However, thoracic skeletal muscle mapping was able to accurately discern IIM from AVM.

## Background

Myocarditis is commonly defined as an inflammatory injury to the myocardium that may result from a variety of causes, most frequently acute viral infection but also systemic inflammatory disease. Idiopathic inflammatory myopathy (IIM) is a group of chronic autoimmune systemic myositis, including polymyositis, dermatomyositis and inclusion body myositis [1]. Clinical presentation includes proximal muscle weakness, myalgia, dysphagia and dyspnea related to respiratory muscle dysfunction and/or interstitial lung disease [2]. Skeletal muscle involvement in IIM may be associated to inflammatory myocardial involvement [3]. When diagnosed, these disorders may be treated with glucocorticoids and immunosuppressive therapy to reduce muscular inflammation and restore muscular performance [4]. Autopsy series have shown histology proven myocarditis to be present in 25% to 30% of patients with IIM [5, 6] and single-photon emission computed tomography (SPECT) studies have demonstrated increased myocardial Technetium-99 m-Pyrophosphate-uptake in 57% of patients with dermatomyositis and polymyositis [7]. Moreover, myocarditis related to IIM requires intensified and prolonged immunosuppression [8] and is the most common cause of adverse outcome and death in IIM [2]. Many occurrences may remain subclinical, but when symptomatic, cardiac symptoms in IIM are similar to acute viral myocarditis (AVM) and include heart failure, arrhythmia and chest pain [9], associated to troponin elevation. In addition, these patients are at increased risk of coronary artery spasm and microvascular disease due to IIM-related vasculitis [10].

Cardiovascular magnetic resonance (CMR) T2-weighted and late gadolinium enhancement (LGE) sequences allow for non-invasive detection of myocarditis in 50% of patients with a troponin-positive episode of chest pain and absence of coronary artery disease. However, the underlying etiology remains unclear in 35% of these cases [11]. New CMR parametric mapping techniques allow to quantify myocardial relaxation times [12] and challenge the original Lake Louise criteria [13], particularly native T1 mapping and extracellular volume fraction (ECV), which were shown to be sensitive markers of myocardial involvement in AVM [14, 15]. Reports of non-invasive characterization of IIM in CMR are scarce. One recent case report showed increased native T1, T2 and T1-derived ECV in one patient with antisynthetase syndrome [16]. Several peripheral muscle investigations were performed in IIM patients using magnetic resonance aimed at edema detection [17], including a few studies with quantitative analysis of peripheral muscle edema using T2 mapping [18].

Our study aims to investigate the usefulness of combined T1 and T2 mapping in cardiac and skeletal muscles as a novel approach to differentiate IIM from AVM in the clinical setting.

## Methods

### Study population

For this retrospective study, a total of 60 participants were included (36 male, 45 ± 16 years): 20 consecutive patients with AVM, 20 consecutive patients with IIM and 20 healthy subjects. AVM patients were included based on clinical guidelines and presented with recent onset chest pain, elevated troponin T and C-reactive protein (CRP), as well as absence of coronary artery disease on angiography, performed in case of acute coronary syndrome-like clinical presentation. Patients without cardiac troponin > 50 ng/ml or with onset of symptoms > 2 weeks before CMR or with prior myocardial infarction were excluded. IIM patients were included based on the elevation of skeletal muscle enzyme levels, evidence of myositis on skeletal muscle biopsy, electromyography (EMG) features of myositis and elevated cardiac troponins > 50 ng/ml at the time of CMR indicating cardiac involvement. Patients with > 2 weeks between troponin elevation and CMR, or with fever or flu-like syndrome in the past 6 months were excluded. Electrocardiogram (ECG) ST-elevation on admission was found in 9/20 of the AVM patients and 4/20 of the IIM patients and led to immediate coronary angiography to assess for coronary artery disease. In addition, 20 asymptomatic subjects without personal medical history or overt cardiovascular disease and normal clinical exam underwent CMR as healthy controls. The study was approved by the local ethics committee and written informed consent was obtained from all participants. The following laboratory test results were collected: hematocrit, NT-proBNP (pg/ml), troponin T (ng/ml), creatine phosphokinase (CPK, IU/l) and CRP(mg/l). The delay between CMR and blood sampling was 4 ± 6 days in IIM patients and 1 ± 1 days in AVM patients. Blood sampling for the control group was performed at the time of CMR. Gender, age, body mass index (BMI) and cardiovascular risk factors were collected. Heart failure was defined by the presence of typical symptoms including breathlessness, fatigue accompanied by signs of elevated right and/or left filling pressures: dilated jugular veins, lower extremity edema, pulmonary edema and/or effusion. Studied IIM patients included: 7 necrotizing autoimmune myopathies, 5 anti-synthetase and 2 overlap syndromes, 3 polymyositis, 2 dermatomyositis and 1 inclusion body myositis.

### CMR protocol

All participants had CMR on a 1.5T magnet (Magnetom Aera, Siemens Healthineers, Erlangen, Germany) with the following acquisition sequences: 1) balanced steady state free precession (bSSFP) cine imaging in short- and long-axis views with typical parameters: acquisition matrix = 216 × 256, repetition time = 51 ms, echo time = 1.19 ms, flip angle = 53°, pixel size = 1.48 × 1.48mm^2^, slice thickness = 6 mm, inter-slice gap = 1 mm. Temporal resolution was between 10 and 40 ms; 2) short- and long-axis LGE sequences acquired with a single shot inversion recovery sequence 7 to 10 min after injection of 0.2 mmol/kg of gadobenate dimeglumine (Multihance, Bracco, Milan, Italy) with the following parameters: acquisition matrix = 240 × 240, inversion time individually chosen on TI scout, repetition time = 347 ms, echo time = 1.18 ms, flip angle = 40°, pixel size = 1.46 × 1.46mm^2^, slice thickness = 8 mm, inter-slice gap = 0.8 mm; 3) Motion-corrected basal, mid-LV and apical short-axis Look-Locker inversion-recovery (MOLLI) T1 mapping sequence with a 5(3)3 scheme before and 15 min after intravenous contrast agent injection with the following parameters: acquisition matrix = 218 × 256, echo time = 1.12 ms, repetition time = 343.86 ms, flip angle = 35°, pixel size = 1.41 × 1.41mm^2^, slice thickness = 8 mm; 4) T2 mapping was performed in basal, mid-LV and apical short-axis slices using a 3-point T2-prepared bSSFP sequence before contrast injection with the following CMR parameters: acquisition matrix = 206 × 256, echo time = 0,24,55 ms, repetition time = 299.74 ms, flip angle = 35°, pixel size = 1.41 × 1.41mm^2^, slice thickness = 8 mm.

### CMR assessment of cardiac volumes, function and LGE

Left ventricular (LV) and right ventricular (RV) volumes and ejection fraction (EF) as well as LV mass have been assessed based on standard short axis semi-automated endocardial and epicardial segmentation. Feature tracking was used to calculate LV and RV myocardial global longitudinal strain (GLS), averaged from 2 chamber and 4 chamber long axis cine views with a dedicated semi-automated software, as previously described [19]. Longitudinal strains were defined as systolic peaks of the temporal curves of myocardial length. For the RV, the lateral wall was used for strain analysis, since the septum is strongly influenced by LV function. The number of segments with LGE were noted for each patient according to the American Heart Association (AHA) 17-segments model.

### CMR mapping

**Fig. 1:**
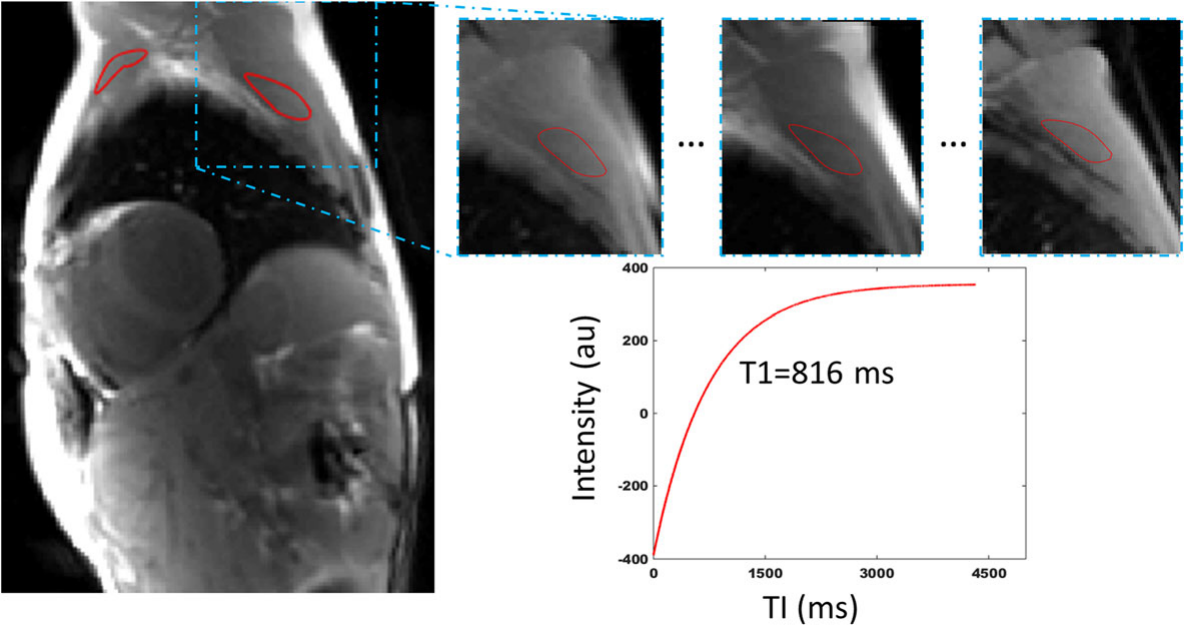
Mapping parameters estimation in the skeletal muscles. As a first step, visible skeletal muscles were delineated on the first TI image after setting the adequate gray levels. Second, each muscle region of interest (ROI) was propagated on all the following TI images. Third, a zoom was performed around each propagated ROIs and gray levels were adjusted to allow their reshaping and displacement in order to avoid fat and vessels as well as to adapt to muscle deformation when necessary. Finally, for each ROI, gray levels were averaged for successive TI images and the resulting signal is fitted with an exponential model to calculate native T1 in the above example. Of note, this process is performed first on native T1 dataset and the resulting ROIs are copied on post-contrast T1 and T2 datasets and the correction process is repeated

Endocardial and epicardial contours were traced with motion-correction for every single TI-image on MOLLI images to exclude near wall blood, epicardial fat or areas presenting Gibbs artifacts. T1 maps were then calculated on basal and mid-LV short-axis slices for both native and post contrast images. Apical slices were not analyzed, due to higher prevalence of motion artifacts and risk of partial volume effect caused by obliquity and thinner wall. Myocardium was automatically segmented into AHA segments using the anterior right septal insertion mark as a reference. Whole-heart myocardial relaxation times were calculated as the mean of mid-LV and basal slices, including and excluding LGE-positive segments. The same analysis was performed for T2 mapping. To estimate thoracic muscle T1 and T2 values, regions of interest (ROI) were traced and adjusted for every TI or TE-image on each of the following skeletal muscles, if identified in the acquired field of view: pectoralis major, subscapularis, infraspinatus, upper arm and erector spinae muscles. The ROI was drawn on the first TI/TE-image by excluding perimuscular fat and intramuscular tendons as illustrated in Fig. 1. For every subsequent TI/TE image, ROIs were modified and eventually shifted in case of muscle movement during image acquisition. Relaxation times were estimated on a per muscle basis and averaged over all muscles for each patient. For both myocardium and skeletal muscles, ECV was calculated as follows [20]:

$$ \mathrm{ECV}=\left(1\hbox{-} \mathrm{hematocrit}\right)\ast \uplambda, \mathrm{with}\ \uplambda =\frac{\frac{1}{\mathrm{T}1\ \mathrm{tissue}\ \mathrm{post}\ \mathrm{contrast}}-\frac{1}{\mathrm{T}1\ \mathrm{tissue}\ \mathrm{native}}}{\frac{1}{\mathrm{T}1\ \mathrm{blood}\ \mathrm{pool}\ \mathrm{post}\ \mathrm{contrast}}-\frac{1}{\mathrm{T}1\ \mathrm{blood}\ \mathrm{pool}\ \mathrm{native}}} $$

### Statistical analysis

Comparisons between IIM and AVM patients and controls were performed using non-parametric Mann-Whitney’s test for continuous variables and Fisher’s Exact Test for categorical variables. For multiple group comparisons (such as T1 and T2 values in different muscle locations), non-parametric Kruskal-Wallis test was used. Receiver operating characteristic (ROC) analysis was used to determine the most discriminating mapping parameter and cutoff values. A *p*-value < 0.05 was regarded as significant. Multivariate analysis was used including age as a covariate to calculate age-adjusted *p*-values for cardiac volumes and mapping indices. Analysis was performed using GraphPad Prism (Version 7.1, GraphPad Software Inc., La Jolla, California, USA) and Stata (Version 11.2, Stata Corporation, College Station, Texas, USA).

## Results

### Patient characteristics

Baseline characteristics of the study population are shown in Table 1. As expected, patients with AVM were younger and mostly males, while IIM patients were older and gender was equally distributed. The mean age of healthy controls fell between the mean age of AVM and IIM patients. AVM and IIM patients had a similar cardiovascular risk profiles except for age. Expectedly, IIM patients had a significantly longer duration of disease (average of 57 months between diagnosis and CMR), while patients with AVM had a shorter duration of disease (average 5 days between first onset of symptoms and CMR). All IIM patients were under anti-inflammatory and immunosuppressive treatment at the time of CMR. CPK was elevated 10× in IIM patients compared with the AVM patients while creatinine was lower in the IIM group due to reduced muscle mass in these patients (normal range 74-107 μmol/l). CRP and NT-proBNP tended to be higher in the AVM group vs. IIM patients although differences did not reach statistical significance (Table 1).

**Table 1:** Baseline Characteristics of the Study Population

	Healthy controls (*n* = 20)		Acute viral myocarditis (*n* = 20)	IIM with inflammatory myocarditis (*n* = 20)	*p* - value
Age, years	47 ± 12		35 ± 13*	54 ± 18	< 0.001
Male / Female	9 / 11		16 / 4*	9 / 11	0.048
BMI, kg/m^2^	25 ± 4		24 ± 6	22 ± 3	0.286
Heart failure	0 (0%)		2 (10%)	1 (5%)	> 0.999
Atrial fibrillation or AV block	0 (0%)		4 (20%)	5 (25%)*	0.725
Significant valvulopathy	0 (0%)		0 (0%)	0 (0%)	
Dyspnea	0 (0%)		2 (10%)	6 (30%)*	0.235
Chest pain	0 (0%)		19 (95%)*	2 (10%)	< 0.001
Dysphagia	0 (0%)		0 (0%)	2 (10%)	0.487
Myalgia	0 (0%)		0 (0%)	7 (35%)*	0.008
Muscle weakness	0 (0%)		0 (0%)	19 (95%)*	< 0.001
Arterial hypertension	0 (0%)		3 (15%)	3 (15%)	
Dyslipidemia	0 (0%)		2 (10%)	5 (25%)	0.408
Diabetes	0 (0%)		0 (0%)	2 (10%)	0.487
Immunosuppressive treatment	0 (0%)		0 (0%)	20 (100%)*	< 0.001
Duration of disease, months	0 ± 0		0.17 ± 0.13	57 ± 53	0.001
NT-proBNP, pg/ml	N/A		1636 ± 3800	699 ± 1226	0.077
Troponin T, ng/ml	N/A		647 ± 610	583 ± 651	0.507
CPK, IU/l	N/A		256 ± 197	2438 ± 3547	0.001
Hematocrit, %	42 ± 3		38 ± 5*	40 ± 3	0.554
Creatinine, μmol/l	82 ± 14		86 ± 53	50 ± 20*	< 0.001
CRP mg/l	1.3 ± 1.3		36 ± 54*	15 ± 25*	0.093

Values are mean ± SD or n (%). *p < .05 compared to controls, *p*-values of the direct comparison between acute viral myocarditis and IIM myocarditis are shown in the last column, using Mann-Whitney U or Fisher’s exact test, as appropriate

*AV* atrio-ventricular, *IIM* idiopathic inflammatory myopathy, *BMI* body-mass-index, *CPK* creatine phosphokinase, *CRP* c-reactive protein, *NT-proBNP* N-terminal pro b-type natriuretic peptide

### Imaging characteristics

Biventricular volumes, functional parameters and strains are reported in Table 2. LVand RV volumes and mass were slightly higher in the AVM group and lower in the IIM group. After age-adjustment, only LV end-systolic volumes remained significantly higher and ejection fractions significantly lower in AVM patients, while only RV end-diastolic volumes remained significantly lower in IIM patients. We found a markedly higher number of patients with LGE, as well as a higher percentage of LGE-positive segments in the AVM group as compared with IIM patients. In addition, IIM patients showed LGE in both epicardial and intramyocardial localizations, while LGE in AVM patients was exclusively epicardial in this study. No LGE was found in controls. While LV global longitudinal strain differentiated controls from IIM patients, no significant differences were found in RV global longitudinal strain.

**Table 2:** CMR Imaging Parameters: ventricular geometry, strain and LGE

	Healthy controls (*n* = 20)	Acute viral myocarditis (*n* = 20)	IIM with inflammatory myocarditis (*n* = 20)	*P*-value / age adjusted *p*-value
LV				
- EDV index, ml/m2	79 ± 16	85 ± 12	78 ± 15	0.059
- ESV index, ml/m2	32 ± 6	40 ± 8*/*	35 ± 13	0.038 / 0.329
- Mass index, ml/m2	54 ± 11	60 ± 10	55 ± 14	0.099
- EF, %	59 ± 4	53 ± 9*/*	56 ± 10	0.198
- GLS, %	−25 ± 3	−24 ± 5	−23 ± 6*	0.235
RV				
- EDV index, ml/m2	98 ± 21	99 ± 21	82 ± 19*/*	0.004 / 0.036
- ESV index, ml/m2	48 ± 14	50 ± 11	40 ± 19*	0.010 / 0.091
- EF, %	51 ± 7	49 ± 4	53 ± 12	0.026 / 0.396
- GLS, %	−32 ± 8	−32 ± 7	−33 ± 9	0.659
LGE				
LGE positive patients	0/20 (0%)	20/20 (100%)*	7/20 (35%)*	< 0.001
Subepicardial LGE positive segments	0/340 (0%)	106/340	9/340 (5%)*	< 0.001
Intramural LGE positive segments	0/340 (0%)	(31%)*	9/340 (3%)*	0.004
Subendocardial LGE positive segments	0/340 (0%)	0/340 (0%)	0/340 (0%)	

Values are mean ± SD or n / total (%). */* *p* < .05 compared to controls without / with age adjustment. *p*-values/age adjusted *p*-values of the direct comparison between acute viral myocarditis and IIM myocarditis are shown in the last column, using Mann-Whitney U and a multivariate regression model or Fisher’s exact test, as appropriate. Age adjusted *p*-values were calculated in case of significant non-adjusted *p*-values

*IIM* idiopathic inflammatory myopathy, *LV* left ventricular, *EDV* end-diastolic volume, *ESV* end-systolic volume, *EF* ejection fraction, *GLS* global longitudinal strain

### Differentiation of AVM and IIM myocarditis

**Fig. 2:**
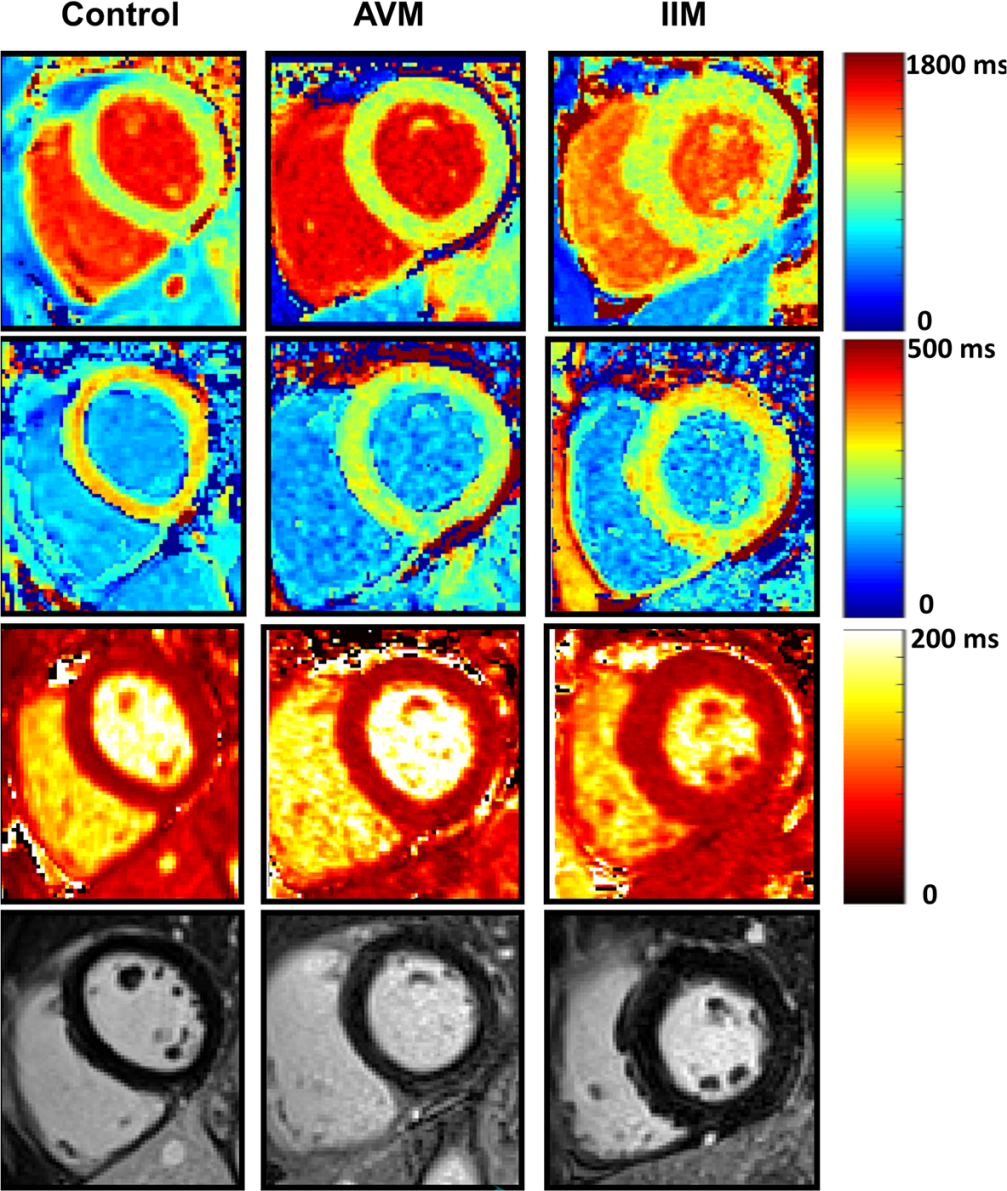
Myocardial T1 and T2 mapping and late gadolinium enhancement (LGE) images. Examples of myocardial T1 mapping in a healthy control subject (female, 33 years old), a patient with AVM (acute viral myocarditis, female, 51 years old) and a patient with IIM (idiopathic inflammatory myositis with cardiac involvement, male, 51 years old). On the first row, native T1 maps are shown (949 ms in the healthy control, 1019 ms in the AVM patient and 1001 ms in the IIM patient. On the second row, post contrast T1 times were measured 332 ms, 277 ms and 306 ms. On the third row, T2 relaxation times were measured 45 ms, 52 ms and 50 ms. Corresponding late gadolinium slices are shown on the last row with slight inferior and infero-lateral sub-epicardial enhancement in the AVM patient, while a minimal infero-lateral subepicardial enhancement can be discussed in the IIM patient. Post contrast T1 in the pectoralis muscle was 469 ms, 499 ms and 399 ms, thus differentiating the IIM patient from the AVM patient and the healthy control

Table 3 shows CMR mapping parameters in the myocardium and in thoracic skeletal muscles. While none of the myocardial mapping parameters was able to differentiate AVM and IIM, irrespective of the inclusion or exclusion of LGE, all skeletal muscle mapping parameters significantly differentiated AVM from IIM. Of note, myocardial mapping parameters significantly separated controls from both groups of patients, even if LGE-positive segments were excluded. These differences remained significant after adjustment for age. Examples of myocardial T1/T2 mapping images in the three groups are shown in Fig. 2.

**Table 3:** CMR Mapping Parameters

	Healthy controls (*n* = 20)	Acute viral myocarditis (*n* = 20)	IIM with inflammatory myocarditis (*n* = 20)	*p*-value / age adjusted *p*-value
Myocardium including LGE positive segments				
T1 native, ms	965 ± 25	1044 ± 63*/*	1017 ± 43*/*	0.121
T1 contrast 15 min, ms	379 ± 54	320 ± 34*/*	326 ± 54*/*	0.925
ECV, %	22 ± 3	24 ± 7*/*	23 ± 3	0.134
T2, ms	48 ± 2	53 ± 4*/*	53 ± 3*/*	0.758
Myocardium excluding LGE positive segment				
T1 native, ms	965 ± 25	1023 ± 52*/*	1011 ± 36*/*	0.445
T1 contrast 15 min, ms	379 ± 54	330 ± 38*/*	328 ± 51*/*	0.718
ECV, %	22 ± 3	25 ± 3*/*	23 ± 4	0.351
T2, ms	48 ± 2	51 ± 3*/*	52 ± 3*/*	0.174
Skeletal Muscles				
T1 native, ms	842 ± 39	844 ± 62	963 ± 127*/*	0.001 / 0.002
T1 contrast 15 min, ms	510 ± 43	482 ± 39*/*	374 ± 55*/*	< 0.001 / < 0.001
ECV, %	10 ± 2	10 ± 3	19 ± 7*/*	< 0.001 / < 0.001
T2, ms	40 ± 2	38 ± 2*	45 ± 10	< 0.001 / 0.022

Values are mean ± SD. */* *p* < .05 compared to controls without / with age adjustment. *p*-values/age adjusted p-values of the direct comparison between acute viral myocarditis and IIM myocarditis are shown in the last column, using Mann-Whitney U test and a multivariate regression model. Age adjusted *p*-values were only calculated in case of significant non-adjusted *p*-values

*IIM* idiopathic inflammatory myopathy, *ECV* extracellular volume fraction, *SD* Standard deviation

**Fig. 3:**
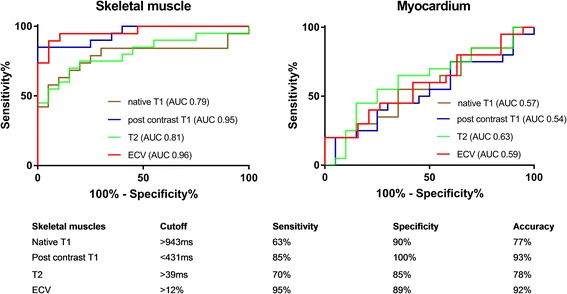
ROC curves illustrating the ability of thoracic skeletal muscles and myocardium mapping parameters to differentiate acute viral myocarditis (AVM) from idiopathic inflammatory myopathy (IIM) myocarditis patients. Area under the ROC curve (AUC) for each mapping parameter are indicated, as well as optimized cutoff values with corresponding sensitivities, specificities and accuracies. ECV = extracellular volume fraction, %

Figure 3 shows ROC curves illustrating the ability of skeletal muscles and myocardium mapping parameters to differentiate IIM from AVM. While skeletal muscle post contrast T1 and ECV performed very well as indicated by a high AUC (0.95 and 0.96 respectively), native T1 and T2 were less useful (AUC 0.79 and 0.81 respectively). In contrast, myocardial mapping parameters were unable to differentiate AVM from IIM (AUC 0.54 and 0.63 respectively). The cutoffs in this study for the detection of myocardial inflammation were > 988 ms for native T1 and > 50 ms for T2, while in the thoracic skeletal muscles, inflammation was best identified with a cutoff of < 431 ms for post contrast T1 and ECV > 12%.

### Comparison of CMR mapping parameters in different muscle groups

**Fig. 4:**
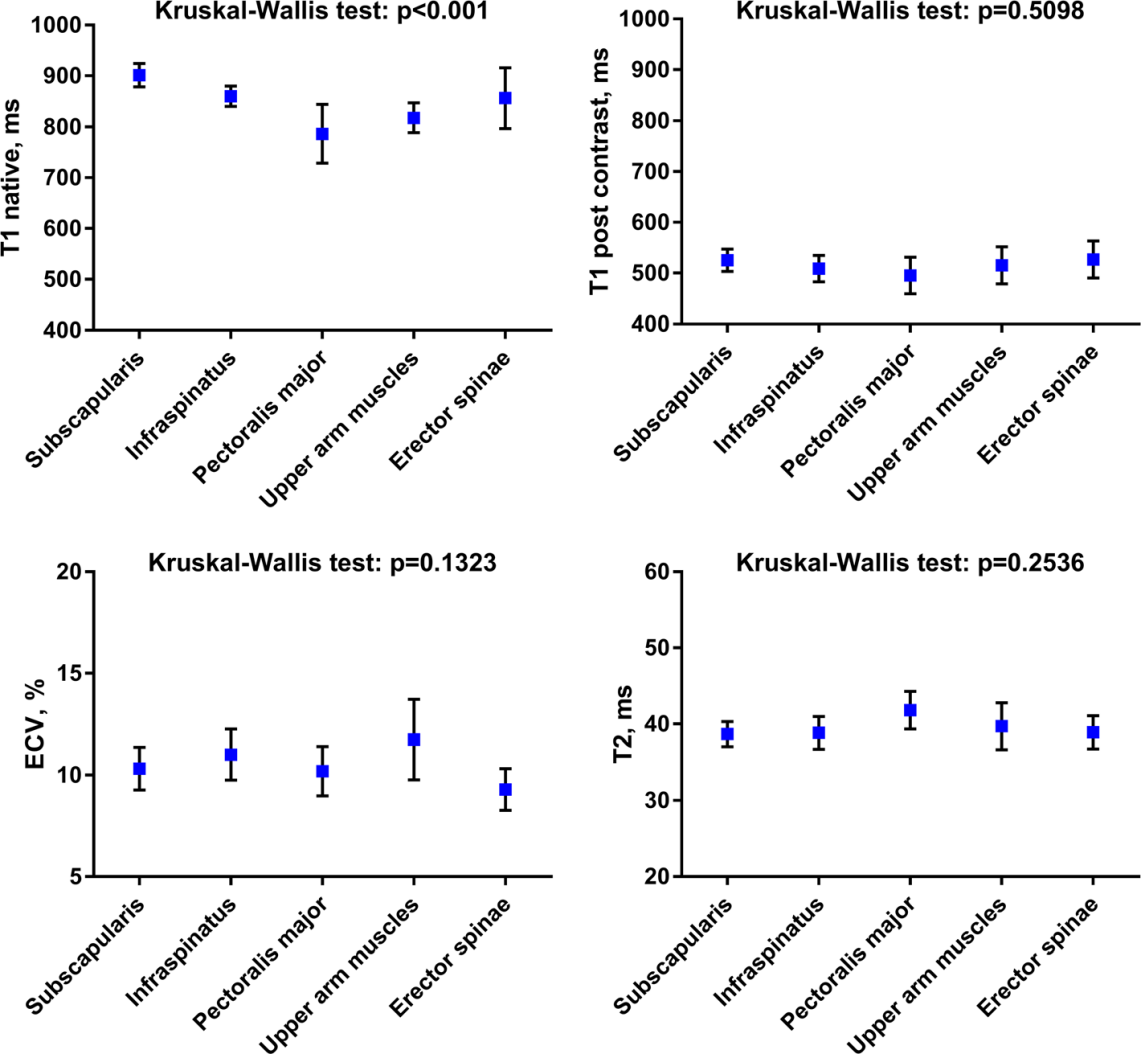
Comparison of different CMR mapping parameters for the measured muscle groups in healthy controls. Error bars indicate 95%-confidence-interval. *P*-value of Kruskal-Wallis test between muscle groups is indicated for each mapping parameter. ECV = extracellular volume fraction, %

Different thoracic muscle groups had similar mapping parameters in healthy controls (Fig. 4), except for native T1, with slightly lower values in the pectoralis major and upper arm muscles and slightly higher values in the subscapularis muscle. The other parameters showed very comparable and robust normal values in all muscles measured in the CMR field of view without significant differences.

## Discussion

This study shows that CMR T1 mapping allows identification of myocardial involvement in IIM without significant differences in myocardial mapping parameters between IIM and AVM. In addition, in unclear cases of suspected myocarditis and CMR with no or few areas of LGE or presence of concomitant muscle weakness, dyspnea or dysphagia, T1 mapping in skeletal thoracic muscle – particularly post contrast T1 and ECV – allow reliable differentiation of acute viral myocarditis and IIM inflammatory myocarditis. To our knowledge, no study had yet compared AVM and IIM myocarditis using both myocardial and skeletal muscle T1 mapping. Yao et al. [18] showed that T2 mapping in muscles of the thigh in IIM patients correlated to clinical severity scores and reported mean T2 values of 60 ms. These T2 values are slightly higher than the ones measured in IIM patients in our study, which might be explained by differences in T2 mapping sequences.

A large number of LGE positive segments were found in the AVM group, corresponding to regions with a high degree of myocardial inflammation and edema. IIM patients also had LGE but less frequently than AVM patients and less frequently in the epicardial layer. Whole heart T1 and T2 mapping parameters were not significantly different between AVM and IIM. However, there was a tendency for higher native T1 in AVM compared with IIM, when LGE positive segments were included, as LGE positive segments are known to result in higher native T1 values compared to LGE-negative segments [21]. Our findings indicate that both AVM and IIM had similar degrees of diffuse myocardial inflammation. Using Lake Louise criteria with LGE as one of the diagnostic criteria for AVM, cases of AVM without LGE might be underdiagnosed to a certain degree in CMR. In relation to our findings, false positives for cardiac involvement may be even more of an issue if such diagnostic criteria are used in the IIM setting. A more widespread use of T1/T2 mapping in clinical routine could help alleviate this issue. Cardiac volumes and mass were higher in the AVM group as compared with the IIM group, which might reflect younger, predominantly male AVM patients compared to probably physically less active IIM patients. Global myocardial strain did not discriminate the two patient groups. Interestingly, all calculated mapping parameters performed very well to differentiate both patient groups from healthy controls, irrespective of the presence of LGE. These findings are in agreement with other T1 mapping studies in AVM patients [14]. Up to our knowledge, the only T1 mapping study to date in IIM with cardiac involvement is one case report showing elevated native T1, ECV and T2 in a patient with antisynthetase syndrome [16].

In addition to IIM, there are other systemic autoimmune diseases with cardiac involvement, such as systemic lupus erythematosus (SLE), sarcoidosis or systemic sclerosis. Native T1 and T2 mapping techniques are able to identify SLE patients from healthy controls [22], which may be useful to detect subclinical myocardial involvement in SLE [23]. Native T1 and ECV are also significantly elevated in sarcoidosis patients without LGE compared to healthy controls, albeit with a notable overlap between the two groups, making the diagnosis of cardiac involvement challenging in the absence of LGE [24]. Myocarditis is also a common finding in SSc, and untreated systemic sclerosis results in myocardial remodeling and fibrosis, as proven by endomyocardial biopsies [25]. Barison et al. [26] showed higher ECV in skeletal muscles of systemic sclerosis patients compared to controls, consistent with extracellular inflammation and remodeling. Further studies combining analysis of myocardial and skeletal muscle relaxation times over the time course of the disease including active and remission phases may help to better characterize such diseases. In addition, AVM associated with skeletal muscle myositis has been reported in cases of influenza B virus infection [27, 28]. However, there are no available data on the combination of myocardial and skeletal muscle T1- and ECV-mapping in such patients necessary to gain more insight into the complex relationship between systemic infection and concomitant myocardial and skeletal muscle inflammation.

Native T1 was significantly different between the skeletal muscle groups, especially in the more peripherally located pectoralis and upper arm muscles. This may have explanations. First, CMR is focused on the heart, using a thoracic coil, and shimming will be less optimized in the periphery of the CMR field-of view. In addition, T1 shortening due to susceptibility might occur, especially in locations close to air near the body surface. Also, possible spill in from peri-muscular fat might occur in patients with thin muscles, affecting especially the pectoralis muscle due to its anatomic localization. These effects may be further aggravated by motion artefacts. Pectoralis muscles may be affected both by breathing and arm movement. Due to shortened T1 after gadolinium injection and due to relative compensation in the ECV formula (ratio), post contrast T1, and ECV were less sensitive to these effects. This may be similar for T2 with refocusing pulses in the SSFP-based sequence. However, post contrast T1, T2 and ECV in different thoracic skeletal muscle groups showed comparable values in healthy controls. Thus, all of the measured muscles can be used for measuring skeletal muscle relaxation time, as visualized on any standard CMR field of view. With these considerations kept in mind and for simplicity, post contrast T1, T2 and ECV may be measured in the best visualized muscle allowing to place the largest ROI excluding vessels, tendons and fat.

**Fig. 5:**
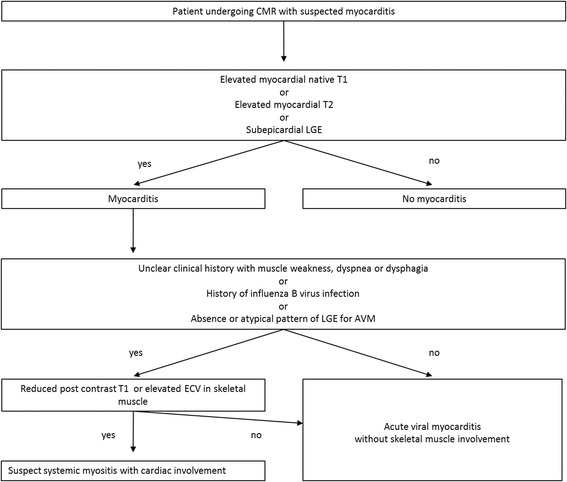
Schematic workflow in patients undergoing CMR for suspected myocarditis. The cutoffs in this study for myocardial inflammation were > 988 ms for native T1 and > 50 ms for T2 in the myocardium, while in the thoracic skeletal muscles, inflammation was best identified with a cutoff of < 431 ms for post contrast T1 and an ECV > 12%. Please note that cutoffs for mapping parameters might significantly vary between centers with different vendors, field strengths and acquisition parameters, so identification of normal values in healthy volunteers is recommended for each CMR magnet [29]. CMR = cardiovascular magnetic resonance; ECV = extracellular volume fraction; IIM = idiopathic inflammatory myopathy

A schematic clinical flow-chart on how mapping techniques might be used in patients with suspected AVM/IIM is proposed in Fig. 5. It should be noted that mapping parameter cutoff values relative to this proof-of-concept study are presented but may not be extrapolated as such to other settings and centers due to important mapping parameter variability as highlighted in recent guidelines [29]. Although IIM-related myocarditis is rare compared to AVM, IIM patients with cardiac involvement need early diagnosis and management, since they have a significantly higher mortality rate compared with IIM patients without cardiac involvement (*p* < 0.001) in longitudinal studies [2]. Consequently, these patients may require intensified immunosuppression [8] unlike AVM patients undergoing more conservative treatment and supportive care [30]. Whether early treatment strategies based on CMR detection of cardiac involvement may improve the prognosis of IIM patients is unknown and warrants further investigation. In addition, further studies should evaluate the utility of T1 and T2 mapping in the skeletal muscle in other systemic diseases such as sarcoidosis, SLE and systemic sclerosis patients.

This study has several limitations. There is a relatively small patient population given the difficulty to recruit IIM patients with elevated cardiac troponins and CMR. Nevertheless, differentiation of acute viral and IIM myocarditis was highly significant using skeletal muscle mapping. Endomyocardial biopsy is not routinely performed in patients with non-complicated myocarditis in our center. In addition, even if all IIM patients had diagnosis proven by skeletal muscle biopsy and histology, there was no such data in the AVM group as there was no indication for biopsy of skeletal muscles in this group. Another limitation is that shimming is not optimized for skeletal muscles in typical CMR exams, potentially affecting native T1 to a higher degree than post contrast T1, ECV and T2 as discussed above. However, the latter three parameters did not differ significantly between different thoracic skeletal muscle groups when peri-muscular fat and intra-muscular tendons were carefully excluded.

## Conclusion

A combined investigation of myocardial and skeletal muscle CMR T1 mapping parameters allow accurate differentiation of AVM from IIM myocarditis by measuring post contrast T1 and ECV in the skeletal muscle. This approach could help in identifying patients with IIM-related cardiac involvement in patients referred to CMR for suspected AVM, allowing differential diagnosis, early IIM treatment and potentially lead to improved patient outcome.

## Abbreviations

AHA: American Heart Association
AVM: Acute viral myocarditis
BMI: Body mass index
bSSFP: Balanced steady state free precession
CMR: Cardiovascular magnetic resonance
CPK: Creatine phosphokinase
ECG: Electrocardiogram
ECV: Extra cellular volume fraction
EDV: End-diastolic volume
EF: Ejection fraction
ESV: End-systolic volume
IIM: Idiopathic inflammatory myopathy
LGE: Late gadolinium enhancement
LV: Left ventricle/left ventricular
MOLLI: Modified Look Locker inversion recovery
ROI: Region of interest
RV: Right ventricle/right ventricular
SLE: Systemic lupus erythematosus
